# Radiation-induced caveolin-1 associated EGFR internalization is linked with nuclear EGFR transport and activation of DNA-PK

**DOI:** 10.1186/1476-4598-7-69

**Published:** 2008-09-12

**Authors:** Klaus Dittmann, Claus Mayer, Rainer Kehlbach, H Peter Rodemann

**Affiliations:** 1 Division of Radiobiology and Environmental Research, Department of Radiation Oncology University of Tübingen, Röntgenweg 11, 72076 Tübingen, Germany; 2 Department of Radiology, Hoppe-Seyler-Str. 3, University of Tübingen, 72076 Tübingen, Germany

## Abstract

**Background:**

To elucidate the role of src kinase in caveolin-1 driven internalization and nuclear transport of EGFR linked to regulation of DNA-repair in irradiated cells.

**Results:**

Ionizing radiation resulted in src kinase stabilization, activation and subsequent src mediated caveolin-1 Y14- and EGFR Y845-phosphorylations. Both phosphorylations were radiation specific and could not be observed after treatment with EGF. Inhibition of EGFR by the antibody Erbitux resulted in a strong accumulation of caveolin/EGFR complexes within the cytoplasm, which could not be further increased by irradiation. Radiation-induced caveolin-1- and EGFR-phosphorylations were associated with nuclear EGFR transport and activation of DNA-PK, as detected by phosphorylation at T2609. Blockage of src activity by the specific inhibitor PP2, decreased nuclear transport of EGFR and inhibited caveolin-1- and DNA-PK-phosphorylation. Knockdown of src by specific siRNA blocked EGFR phosphorylation at Y845, phosphorylation of caveolin-1 at Y14 and abolished EGFR transport into the nucleus and phosphorylation of DNA-PK. Consequently, both knockdown of src by specific siRNA and also inhibition of src activity by PP2 resulted in an enhanced residual DNA-damage as quantified 24 h after irradiation and increased radiosensitivity.

**Conclusion:**

Src kinase activation following irradiation triggered caveolin-1 dependent EGFR internalization into caveolae. Subsequently EGFR shuttled into the nucleus. As a consequence, inhibition of internalization and nuclear transport of EGFR blocked radiation-induced phosphorylation of DNA-PK and hampered repair of radiation-induced double strand breaks.

## Background

Many human tumor cells are characterized by over-expression of epidermal growth factor receptor (EGFR), a protein that promotes growth and aggressiveness and resistance of cancer cells to chemo- and radiotherapy [1-5]. EGFR can be phosphorylated in response to binding of its specific ligands (EGF, TGF alpha and Amphiregulin) [6,7] and after exposure to unspecific stimuli like ionizing radiation [8], UV-radiation [9], hypoxia [10], hyperthermia [11], oxidative stress [12] and trans-activation by G-protein coupled receptors [13,14]. Ligand-dependent as well as ligand-independent phosphorylation of EGFR results in receptor internalization [15] and intracellular signaling [4,5,16-18]. Up to date internalization is assumed to be essential for receptor silencing and inactivation. Indeed, EGF treatment results in internalization of EGFR into coated pits followed by receptor degradation [19]. As reported by Khan [12], exposure to oxidative stress can lead to internalization of EGFR by caveolae and this process is associated with peri-nuclear accumulation of EGFR.

A characteristic constituent of caveolae is caveolin. In vertebrates the caveolin gene family has three members: CAV1, CAV2, and CAV3, coding for the proteins caveolin-1, caveolin-2 and caveolin-3, respectively. Caveolins form oligomers and associate with cholesterol and sphingolipids in certain areas of the cell membrane, leading to the formation of caveolae. Caveolae are involved in receptor independent endocytosis [20]. Furthermore Caveolin-1 is an integral transmembrane protein and an essential component in interactions of integrin receptors with cytoskeleton-associated and signaling molecules [21]. Compartmentation into caveolae prevents EGFR degradation and simultaneously enables intracellular EGFR signaling [12]. These findings suggest a new function of EGFR – depending on its intracellular localization -, which supplements its functions described so far. The idea of additional EGFR functions is further supported by the observation, that peri-nuclear EGFR can be transported into cell nucleus in response to irradiation [5]. As we and others have reported earlier [4,22-24], nuclear EGFR is linked with activation of DNA-PK and regulation of non-homologous end-joining DNA-repair resulting in increased radioresistance [5]. As reported recently [1], nuclear EGFR detection in tumors biopsies correlated strongly with treatment resistance and bad prognosis.

In the present study, we focused on the radiation-induced nuclear translocation process of EGFR via caveolae. Evidence is provided that inhibition of src activity blocks the caveolin-dependent EGFR internalization and nuclear EGFR transport, which results in impaired DNA-repair.

## Materials and methods

### Cell culture, transfection, irradiation and colony formation assay

Experiments were performed with the human bronchial carcinoma cell line, A549 (ATCC) and the human squamous carcinoma cell line FaDu (ATCC, origin head and neck cancer). Cells were irradiated with 200-kV photons (Gulmay RS 225, dose rate 1 Gy/min) at 37°C. The EGFR-inhibitory antibody Erbitux was purchased from Merck KG aA, Germany and was administered to the cells at a concentration of 30 nM 1 h before irradiation. PP2 (4-amino-5-(4-chlorophenyl)-7-(*t*-butyl)pyrazolo [3,4-*d*]pyrimidine) was received from Sigma and cells were treated at a concentration of 100 nM PP2 dissolved in DMSO for 1 h. For silencing of src cells were treated with specific siRNA for 72 hours before irradiation. Control non-silencing siRNA (sense UUCUCCGAACGUGUCACGUtt; antisense ACGUGACACGU-UCGGAGAAtt) and siRNA targeting src (sense ACUCGCCUUCUUAGAGUUUtt; antisense AAACUCUAAGAAGGCGAGUtt) probes were purchased from MWG-Biotech AG. Both were transfected at a concentration of 30 nM using Lipofectamine 2000 transfection reagent according to manufacture's protocol (Invitrogen). For colony formation assay cells were grown to confluence, treated as indicated and irradiated. After 6 hours cells were typsinized and seeded at a density of 500 cells in 78 cm^2 ^plates. After 10 days colonies were fixed, stained and counted. Radiation survival curves were plotted after normalizing for the cytotoxicity induced by siRNA treatment or vehicle alone. Clonogenic survival curves were constructed from at least three independent experiments.

### Subcellular fractionation

Cytoplasmic and nuclear extracts were prepared according to the instructions of the NE-PER^® ^nuclear and cytoplasmic extraction kit (Pierce, Rockford, IL, USA).

### Western blot analysis and immune-precipitation

After irradiation, as described above, cells were lysed and proteins were resolved by SDS-PAGE. Western blotting was performed according to standard procedures [25]. The primary antibodies were diluted as follows: anti-EGFR (BD Transduction Laboratories, clone 13) 1:1000; anti EGFR pY845 (nanotools, clone 12A3) 1:1000; anti-EGFR pY992 (abcam, polyclonal) 1:500); anti-EGFR pY1173 (Cell signaling, clone 53A5) 1:1000; anti-phosphotyrosin (Santa Cruz, clone PY20) 1:500); anti-src (Santa Cruz, clone H-12) 1:1000; anti-src Y416 (cell signaling, polyclonal), 1:1000; anti-caveolin-1 (BD Transduction Laboratories, clone 2297) 1:1000; anti-caveolin pY14 (BD Transduction Laboratories, clone 56) 1:1000; anti-DNA-PK (PharMingen, clone 4F10C5) 1:500; anti-DNA-PK pT2609 (Rockland) 1:1000; anti-lamin B1 (Biozol, clone ZL-5) 1:1000. Quantification of binding was achieved by incubation with a secondary peroxidase-conjugated antibody with the ECL system (Amersham).

EGFR was immune-precipitated from cytoplasmic and nuclear protein fractions prepared from 20 × 10^6 ^cells with EGFR antibody clone 13 (BD Transduction Laboratories). Immune-precipitation was performed as described [26].

### Quantification of γH_2_AX-foci formation

Cells cultured on CultureSlides (Becton Dickinson) were incubated with PP2 or src-siRNA, irradiated and fixed with 70% ice-cold ethanol 24 h after irradiation. For immune-fluorescence analysis cells were incubated with **γ**H_2_AX antibody (Upstate, clone JBW301)(1:500) at room temperature for 2 h. Positive foci were visualized by incubation with a 1:500 dilution of Alexa488-labelled goat anti-mouse serum (Molecular Probes) for 30 min. Coverslips were mounted in Vectashield/DAPI (Vector Laboratories). For each data point 300 to 500 nuclei were evaluated.

## Results

### Caveolin-1 associated EGFR internalization following irradiation was triggered by src kinase

**Figure 1:**
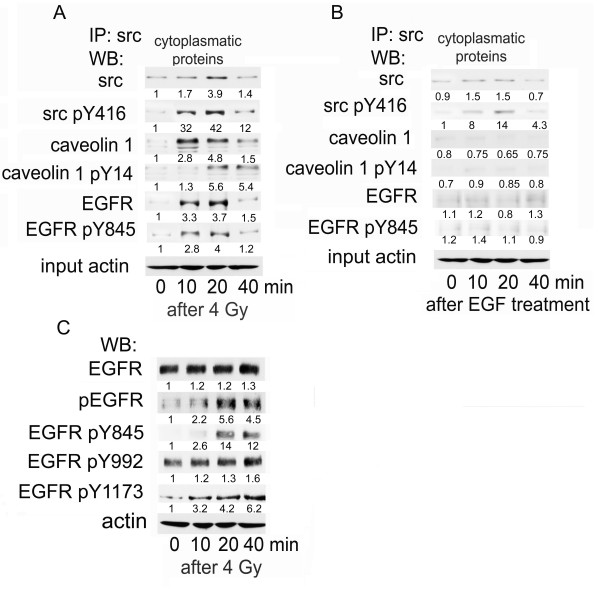
**Radiation induced caveolin-1 linked EGFR internalization is triggered by src kinase**. Confluent A549 cells were irradiated with 4 Gy (A). At the time points given, src protein was immune-precipitated from cytoplasmic protein fraction. Proteins were separated by SDS-PAGE and after blotting the protein amounts of src, caveolin1 and EGFR were quantified. The same procedure was applied after cell stimulation with EGF (B). Phosphorylation of EGFR at the tyrosine No. 845, 992 and 1173 was quantified with help of specific antibodies following a standard western procedure. Equal protein amounts for immune-precipitation were documented by showing actin expression within aliquots of input proteins. (C). Protein expression was quantified by densitometry and shown as fold-induction relative to untreated control. The mean protein expression derived from three experiments was given below each band.

Ionizing radiation induced protein stabilization of cytoplasmic src in A549 cells within 10 to 20 min (Fig. 1A). Stabilization of src was associated with phosphorylation at residue Y416, which indicates src activation [27]. Radiation-induced stabilization and activation of src led to complex formation with caveolin-1 and EGFR as shown by means of a src-specific immune-precipitation. Caveolin-1 associated EGFR was phosphorylated at residue Y845, which is a known src kinase specific phosphorylation site [28]. Moreover, increased src activity was associated with increased caveolin-1 and EGFR binding and phosphorylation at Y14 and respectively Y845 (Fig. 1A). EGF treatment also stabilized src protein and triggered a weaker Y416 phosphorylation. However, caveolin-1 and EGFR were not increased over the control in the src associated complex following EGF treatment (Fig. 1B). To elucidate the role of src-driven EGFR phosphorylation at residue Y845 following irradiation, we determined the phosphorylation status of additional tyrosine residues of EGFR after irradiation with 4 Gy (Fig. 1C). In general, tyrosine phoshorylation of EGFR was markedly increased. Especially phosphorylation at residues Y845 and Y1173 were strongly induced by irradiation, whereas the induction of phosphorylation at residue Y992 was weaker.

### The antibody Erbitux stabilized the cytoplasmic caveolin-1/EGFR complex

**Figure 2:**
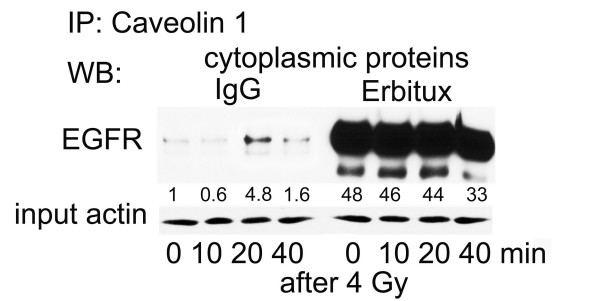
**Stabilization of caveolin1/EGFR complex in cytoplasm after incubation with EGFR inhibitory antibody Erbitux**. Confluent A549 cells were treated with human IgG or EGFR inhibitory antibody Erbitux at a concentration of 30 nM for 1 h. Subsequently cells were irradiated with 4 Gy and cytoplasmic and nuclear proteins were isolated at time points given. Proteins were separated by SDS-PAGE and after blotting EGFR protein was quantified with help of a specific antibody. Experiments were performed three times; shown are representative results. Equal protein input for immune-precipitation was documented by showing actin expression within aliquots of input proteins. Expression of specific proteins was quantified by densitometry and shown as fold-induction relative to untreated control.

In agreement with Fig. 1, immune-precipitation targeting caveolin-1 indicated also an increasing complex formation with EGFR following radiation exposure in A549 cells (Fig. 2). Pre-incubation (1 h) with the EGFR inhibitory antibody Erbitux, resulted in a pronounced stabilization of the caveolin-1/EGFR complex in non-irradiated cells. This complex formation could not be further increased by radiation exposure.

### Src kinase inhibitor PP2 prevented radiation-induced EGFR transport into the nucleus and hampered radiation-induced activation of DNA-PK

**Figure 3:**
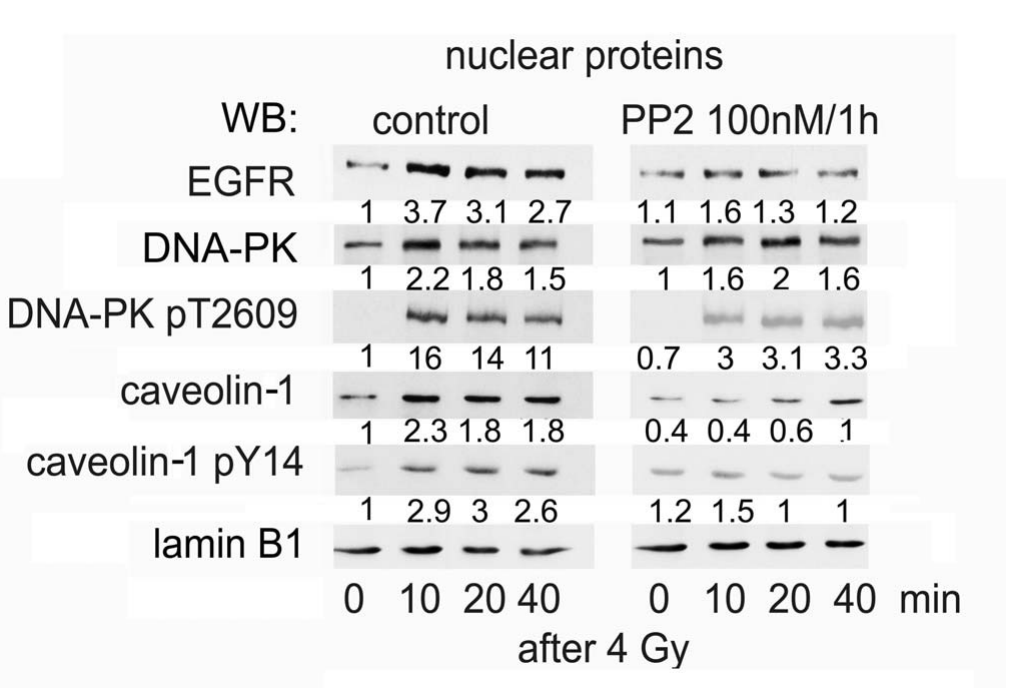
**Inhibition of nuclear EGFR transport and phosphorylation of DNA-PK by src inhibitor PP2**. Confluent A549 cells were treated with src inhibitor PP2 at a concentration of 100 nM for 1 h. Subsequently cells were irradiated with 4 Gy and nuclear proteins were isolated at the time points given. Proteins were separated by SDS-PAGE and after blotting protein amounts were quantified by help of specific antibodies. Expression of specific proteins was quantified by densitometry, normalized to lamin B1 and shown as fold-induction relative to untreated control. Experiments were performed three times; shown are representative results.

As already shown earlier [5], ionizing radiation triggered EGFR transport into the nucleus and complex formation with DNA-PK (Fig. 3) [5]. Treatment with the src kinase inhibitor PP2 (100 nM for 1 h) was sufficient to block radiation-induced nuclear translocation of EGFR in A549 cells (Fig. 3). Interestingly, basal amount of nuclear EGFR protein was not affected by PP2 treatment. However, total nuclear caveolin-1 protein and its phosphorylated form was decreased by PP2 treatment. In addition, phosphorylation of DNA-PK at residue T2609, which is essential for non-homologous end-joining DNA-repair process [29], was inhibited in response to PP2 incubation, whereas the amount of total DNA-PK protein was unchanged.

### Src siRNA decreased phosphorylation of cytoplasmic EGFR at Y845, reduced EGFR transport into nucleus and impaired phosphorylation of DNA-PK at T2609 after irradiation

**Figure 4:**
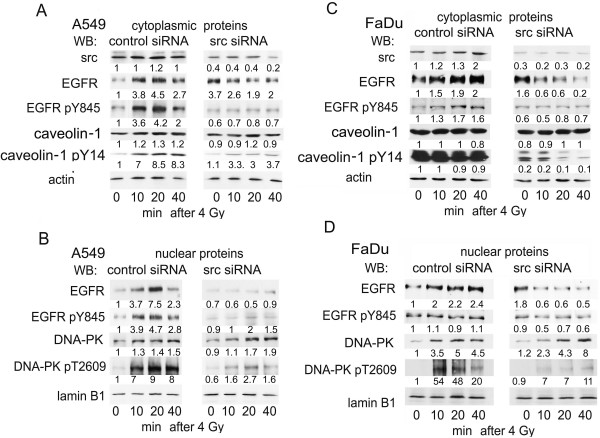
**Inhibition of EGFR transport into the cytoplasm/nucleus by src siRNA**. Exponentially growing A549 cells were incubated either with control- or src-specific siRNA for 72 h at a concentration of 30 nM. Subsequently confluent cells were irradiated with 4 Gy and cytoplasmic (A) and nuclear proteins (B) were isolated at the time points given. Corresponding results were presented for FaDu cells (C and D). Expression of specific proteins was quantified by densitometry, normalized either to actin or lamin B1 and shown as fold-induction relative to untreated control. Experiments were performed three times; shown are representative results.

Src protein expression was effectively knocked down in A549 and FaDu cells 72 h after transfection with src specific siRNA (Fig. 4A/C). Repression of src-protein markedly inhibited radiation-induced phosphorylation of cytoplasmic EGFR at Y845 and caveolin Y14 (Fig. 4A/C) after irradiation. In agreement with the data of Fig. 3, src knockdown also reduced radiation-induced EGFR shuttling into nucleus (Fig. 4B/D). Moreover, like PP2 inhibitor (see Fig. 3) src siRNA treatment led to reduced phosphorylation of DNA-PK at residue T2609 (Fig. 4B). It is noteworthy, that basal amount of nuclear EGFR protein was increased already by src siRNA treatment alone. However, this increase did not correspond to an activation/phosphorylation of DNA-PK at T2609.

### Blocking of src signaling increased level of residual DNA-damage following irradiation

**Figure 5:**
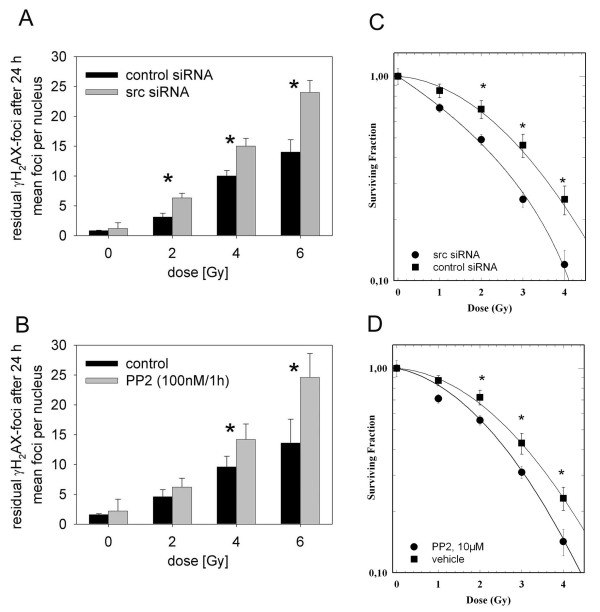
**Inhibition of DNA-repair and clonogenic survival by src siRNA or src inhibitor PP2**. A549 cells were either treated with src siRNA (72 h) (A/C) or with src inhibitor PP2 (1 h) (B/D). Subsequently cells were irradiated with 2, 4 and 6 Gy and after 24 h cells were fixed. Residual damage was visualized by incubation with γH_2_AX antibody (A/B). Each bar represents the mean ± SE of residual repair foci positive for γH_2_AX per cell nucleus. For each data point 300 nuclei were evaluated. Asterisks indicate significant differences (Student's t-test * p < 0.05). For colony formation assay cells pretreated either with src siRNA (C) or PP2 (D) were irradiated and seeded at a density of 500 cells per 78 cm^2 ^dish. After 10 days colonies were fixed and stained. Surviving fractions were calculated on the basis of colony counts and plating efficiency. Each value represents the mean of three independent experiments. Differences were considered as statistically significant for p < 0.05 (t-test) and were marked with an asterisk *.

Irradiation of confluent A549 cells treated with control siRNA resulted in a radiation- dose dependent increase in residual γH_2_AX positive repair foci, which represent un-repaired DNA double strand breaks (Fig. 5A). Pre-treatment with src specific siRNA increased the number of radiation-induced residual γH_2_AX foci 24 h after irradiation, by a factor of 1.5 – 2 (Fig. 5A). Likewise pre-treatment of cells with the src kinase inhibitor PP2 for 1 h, resulted also in a significant increase in residual γH_2_AX foci (Fig. 5B). In both cases inhibition of DNA-damage repair was correlated with increased radiosensitivity, as determined by means of colony formation assay (Fig. 5C/D).

## Discussion

It is generally accepted, that the epidermal growth factor receptor is localized within the cell membrane and will be internalized following activation and dimerization [30]. Indeed, such a scenario can be observed following EGF stimulation [30], which initiates proliferation associated signaling. However, the EGFR can be also activated by oxidative stress [12], radiation, [5,8] and G-coupled receptors [13]. The molecular mechanisms of this ligand independent activation of EGFR are not fully understood. However, ligand independent stimulation of EGFR, e.g. by ionizing radiation [8], is clearly characterized by receptor internalization also. The data presented herein, give new insights into the mechanism of EGFR internalization process and the intra-nuclear function of EGFR following exposure to ionizing radiation.

Several pathways enable endocytic transport of cargo molecules from the surface of eukaryotic cells into cytoplasm [31]. The two best understood pathways, relevant for EGFR internalization, are the clathrin-coated pit [31] and the caveolin [32] driven internalization mechanisms. As shown by Khan et al. [12], the clathrin-coated pit associated EGFR internalization can be observed following treatment with EGF and results in a fast degradation and silencing of receptor function. In contrast, treatment with H_2_O_2 _leads to EGFR internalization into caveolae, which sort internalized EGFR into a per-nuclear localization associated with an ongoing receptor signaling [12]. In agreement with these data, we could show, that exposure to ionizing radiation induced a caveolin-1 associated EGFR internalization, whereas EGF treatment failed to trigger complex formation between *src*, EGFR and caveolin-1. Sorting into different compartments in response to different stimuli may explain signal discrimination at the level of activated EGFR. Like for H_2_O_2 _treatment [12], exposure to ionizing radiation also mediates the src driven phosphorylations of EGFR at Y845 and of caveolin-1 at Y14, which is needed for internalization of EGFR into caveolae [12]. In response to radiation not only EGFR phosphorylation at Y845 – which is Src dependent – was observed, but also phosphorylation at Y992 and Y1173 could be observed. Both are described as autophosphorylation sites [33]. This implicates that ionizing radiation activates not only src kinase, but also EGFR kinase and both kinases contribute to altered phosphorylation pattern of EGFR following radiation exposure. Caveolin-1 phosphorylation seems to be critical for caveolae formation [20]. On the contrary, Y845 phosphorylation of EGFR probably is rather essential in regulation of EGFR-kinase activity than in formation of coated pits or caveolae [15]. However, as shown by us and by Khan [12] src activity is crucial for radiation- and H_2_O_2_-induced formation of caveolae. Nevertheless, the molecular mechanism responsible for activation of src has to be resolved. From our data it appears, that radiation leads to a fast activation of src, which is documented by phosphorylation of src at residue Y416. This phosphorylation is described as an autophosphorylation [27]. As activating molecular switch several mechanisms are discussed: (i) oxidation associated structural modifications result in activation of src kinase [34], (ii) inhibition of a phosphatase leads to auto-activation of kinase [35], (iii) G-coupled receptor signaling mediates src activation [14]. Which of these potential mechanisms is relevant for radiation-induced src kinase activity is currently unclear and is subject of ongoing investigations.

As shown herein, treatment with Erbitux, which binds to the extracellular domain of EGFR, results in receptor internalization and formation of an intracellular complex of EGFR, caveolin-1 and Erbitux. Internalized EGFR however can not be activated by EGF and this observation may explain growth inhibitory effects of Erbitux.

Khan et al. observed a peri-nuclear EGFR accumulation due to caveolin-1 driven internalization after exposing cells to H_2_O_2 _[12]. We could also detect a peri-nuclear localization of the EGFR [5] in irradiated cells, which is accompanied by a nuclear EGFR shuttling [5]. Based on these results we hypothesized, that peri-nuclear EGFR serves as a pool for nuclear EGFR transport following irradiation. This hypothesis is supported by the observation that inhibition of src either by its specific inhibitor PP2 or by specific siRNA, prevents nuclear translocation of EGFR by blocking caveolin-1 driven EGFR internalization. It is noteworthy, that caveolin-1 driven EGFR internalization occurs predominantly following treatment of cells with genotoxic agents. This observation is in favor with the idea, that EGFR internalization and nuclear transport of EGFR are linked with DNA-repair processes [23,36]. This assumption is supported by the observation, that caveolin-1 driven EGFR internalization is not observed after EGF treatment. As shown for irradiated cells nuclear EGFR is found in complex with DNA-PK, which is an essential compound of non-homologous end-joining DNA-repair [5]. As reported earlier [37], inhibition of EGFR nuclear transport by Erbitux, markedly impaired radiation associated activation of DNA-PK and increased cellular radiosensitivity [37]. In agreement with that, inhibition of src, which blocks EGFR internalization and subsequently nuclear transport after irradiation, abolished activation of DNA-PK, inhibited DNA-repair and increased radiosensitvity. Based on the data presented, it can be concluded, that the radiation-induced activation and nuclear translocation of EGFR is mediated through src kinase activity in a caveolin-1 dependent process. As blocking of these processes markedly effects repair of DNA-double strand breaks, this EGFR-coupled radiation response mechanism offers new interventional molecular targets for cancer therapy, especially by radiation therapy.

## Conclusion

EGFR internalization by caveolin-1 is a stress specific cellular reaction, which is src kinase activity dependent. Linked with EGFR internalization nuclear transport can be observed following irradiation. Nuclear EGFR transport can be hampered by inhibition of src. Consequently, src inhibition is associated with inhibition of EGFR triggered activation of DNA-PK, which leads to an inhibition of DNA-repair and cell survival.

## Competing interests

The authors declare that they have no competing interests.

## Authors' contributions

KD, CM and RK performed experiments and interpreted data; the authors contribution to this research are reflected in the order shown. HPR supervised all aspects of this research. KD and HPR prepared the manuscript. All authors read and approved the final manuscript.
